# Mapping the FACT-B Instrument to EQ-5D-3L in Patients with Breast Cancer Using Adjusted Limited Dependent Variable Mixture Models versus Response Mapping

**DOI:** 10.1016/j.jval.2018.06.006

**Published:** 2018-12

**Authors:** Laura A. Gray, Allan J. Wailoo, Monica Hernandez Alava

**Affiliations:** Health Economics and Decision Science, School of Health and Related Research, University of Sheffield, Sheffield, South Yorkshire, UK

**Keywords:** mixture models, utility mapping, EQ-5D-3L, ALDVMM, FACT-B

## Abstract

**Background:**

Preference-based measures of health, such as the three-level EuroQol five-dimensional questionnaire (EQ-5D-3L), are required to calculate quality-adjusted life-years for use in cost-effectiveness analysis, but are often not recorded in clinical studies. In these cases, mapping can be used to estimate preference-based measures.

**Objectives:**

To model the relationship between the EQ-5D-3L and the Functional Assessment of Cancer Therapy—Breast Cancer (FACT-B) instrument, comparing indirect and direct mapping methods, and the use of FACT-B summary score versus FACT-B subscale scores.

**Methods:**

We used data from three clinical studies for advanced breast cancer providing 11,958 observations with full information on FACT-B and the EQ-5D-3L. We compared direct mapping using adjusted limited dependent variable mixture models (ALDVMMs) with indirect mapping using seemingly unrelated ordered probit models. The EQ-5D-3L was estimated as a function of FACT-B and other patient-related covariates.

**Results:**

The use of FACT-B subscale scores was better than using the total FACT-B score. A good fit to the observed data was observed across the entire range of disease severity in all models. ALDVMMs outperformed the indirect mapping. The breast cancer–specific scale had a strong influence in predicting the pain/discomfort and self-care dimensions of the EQ-5D-3L.

**Conclusions:**

This article adds to the growing literature that demonstrates the performance of the ALDVMM method for mapping. Regardless of which model is used, the subscales of FACT-B should be included as independent variables wherever possible. The breast cancer–specific subscale of FACT-B is important in predicting the EQ-5D-3L. This suggests that generic cancer measures should not be used for utility mapping in patients with breast cancer.

## Introduction

Outcomes recorded in clinical studies often do not include preference-based measures (PBMs) such as the three-level EuroQol five-dimensional questionnaire (EQ-5D-3L). Nevertheless, PBMs are required for use in cost-effectiveness analysis when quality-adjusted life-years need to be calculated. Health utility mapping is used to estimate a PBM using other outcomes that have been collected in clinical trials, including clinical outcome measures. In cancer studies there is often a focus on survival time and on disease-specific quality-of-life measures. This article aims to develop a mapping algorithm that estimates EQ-5D-3L scores in patients with breast cancer.

The EQ-5D-3L is one of the most widely used PBMs, comprising five dimensions (mobility, self-care, usual activities, pain/discomfort, and anxiety/depression) with three levels per dimension (no/moderate/extreme problems).

The Functional Assessment of Cancer Therapy—Breast (FACT-B) [1] is a self-reported instrument that measures health-related quality of life of patients with breast cancer. It comprises five subscales including physical well-being, social well-being, emotional well-being, functional well-being, and a breast cancer–specific (BCS) subscale. Each subscale has seven items, with the exception of the emotional well-being subscale, which has six items, and the BCS subscale, which has nine items. Items are rated from 0 (not at all) to 4 (very much) and a total score is derived. The total FACT-B score ranges from 0 to 123, and is calculated by adding the scores from each of these subscales. Lower scores indicate better health.

The only previous mapping study using FACT-B, according to the Health Economics Research Centre Database of Mapping Studies [2], mapped to the five-level EQ-5D using data from Singapore and applied only simple linear regression methods. There is a large literature that shows that the theoretical assumptions of linear regression methods are not met when mapping to the EQ-5D-3L and that they produce biased results [3], [4]. Response mapping has been shown to outperform standard statistical techniques such as linear regression, generalized linear models, and fractional logistic models [5]. Mixture models, however, have previously been shown to outperform both linear regression and response mapping in a range of settings [4], [6], [7]. Mapping models that use the individual subscale scores of an instrument to map to a PBM are expected to have a model fit superior to those that use only the total instrument score; condensing outcome data into a summary measure discards important information but may be more practically relevant to those wishing to use the mapping results in economic models that rely on data that include the summary score only.

In this article we map from the FACT-B instrument to the EQ-5D-3L, using bespoke adjusted limited dependent variable mixture models (ALDVMMs) [8] and compare results to response mapping using seeming unrelated (SUR) ordered probit models. We also compare both techniques using the total FACT-B score and the separate subscale scores of the FACT-B.

## Methods

### Data

Three phase III clinical studies measured both the EQ-5D-3L and FACT-B and were available for analysis.

The Treatment Across multiple liNes wIth Avastin (TANIA) trial recruited patients with HER2-negative locally recurrent/metastatic breast cancer whose disease had progressed on or after first-line bevacizumab combined with chemotherapy. They were randomized to receive standard second-line chemotherapy either alone or in combination with bevacizumab [9]. In total, 494 patients were randomized. FACT-B was administered at baseline, every 8 or 9 weeks (depending on treatment schedule) during second-line therapy, and at the time of second progression. The EQ-5D-3L was administered at baseline, during second-line treatment at weeks 8 and 16 (4-week cycles) or at weeks 9 and 18 (3-week cycles), and at second-line disease progression (up to ~3 years).

The MARIANNE study is an international, randomized, multicenter, three-arm study involving 1095 people with HER2-positive advanced breast cancer. Patients were assigned 1:1:1 to control (trastuzumab plus taxane), T-DM1 plus placebo, and T-DM1 plus pertuzumab at standard doses.

The Batman study was an open-label, single-arm, multicenter UK study of the safety and tolerability of bevacizumab when combined with taxane monotherapy as first-line therapy of 50 patients with triple-negative metastatic breast cancer.

Data were pooled to maximize sample size and reduce uncertainty.

### Statistical Analysis

The EQ-5D-3L has a distinctive distribution. Here, we used the UK tariff but its characteristics are common to many other countries. It is bounded below at −0.594 and above at 1. It includes a multimodal distribution as well as a mass of observations at 1, representing full health. There is a gap between full health and the next feasible health state (0.883). These properties are difficult to estimate using standard statistical techniques. We used two statistical methods in an attempt to overcome this problem.

First, we applied response mapping by using SUR ordered probit models using the “cmd” command in Stata (StataCorp, College Station, TX) for estimating conditional mixed process models with multilevel random effects and coefficients. This method estimates responses to each of the five EQ-5D-3L dimensions, jointly using ordered probit models allowing the error terms to be correlated. We then calculated the expected EQ-5D-3L scores on the basis of the probabilities of each of the possible 243 health states as a separate second step.

Second, we used ALDVMMs using the publicly available Stata command “aldvmm” [8]. These bespoke models were specifically designed for mapping to the EQ-5D-3L. These include a discrete element using multinomial logit models for the probability of component (or latent class) membership, including a component representing full health. Using multiple components in a mixture model allows us to capture the multimodal properties of the distribution. The EQ-5D-3L does not show (trastuzumab emtansine) a normal distribution and so it is important to be able to accurately estimate the unusual distribution. By limiting the dependent variable, the ALDVMM prevents prediction outside the feasible range: it cannot predict below −0.594 or above 1. It also takes into account the unfeasible gap between full health and the next feasible health state. Independent variables can influence component membership and/or each of the components in the mixture model part. This model has been used with success in previous mapping studies [4], [6], [7], [10], [11], [12].

The FACT-B score is made up of a set of subscores: physical, social, emotional, and functional well-being and a BCS subscale. All cancer-specific FACT-B scores include the first four of these subscores as well as a cancer-specific subscale. We mapped from the FACT-B instrument to the EQ-5D-3L using two sets of covariates. First, we used the total FACT-B score, the age of the patient, and age-squared. Second, we used the individual subscores of the FACT-B, the age of the patient, and age-squared. Models were estimated without age included, but age was found to improve the fit of the models. We did not include sex because there were very few male patients in the data set. We used the UK value sets for the EQ-5D-3L [13].

To compare results across models we considered different measures of model fit in line with the International Society for Pharmacoeconomics and Outcomes Research Good Practice Guide on Mapping [14]. To determine the preferred number of components in the mixture models, we considered model fit using mean absolute error (MAE), root mean square error (RMSE), and Akaike and Bayesian information criteria (AIC and BIC, respectively) as well as considered visual representations of model fit. We plotted the mean of the predicted values of FACT-B with the mean of the observed values, for the entire range of disease severity. We also simulated data from the models and plotted the cumulative distribution functions (CDFs), comparing simulated with observed data across the spectrum of disease severity. These CDFs are an important means of assessing model fit relevant to those settings that may use the mapping for analysis at the patient level, for example, in a trial-based cost-effectiveness analysis or a patient-level decision model [14].

## Results

**Fig. 1:**
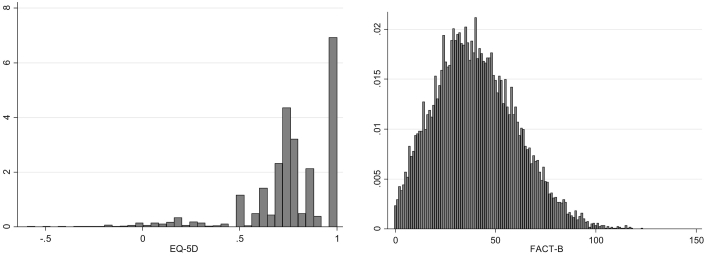
EQ-5D-3L and FACT-B distributions. EQ-5D-3L, three-level EuroQol five-dimensional questionnaire; FACT-B, Functional Assessment of Cancer Therapy—Breast Cancer.

The combined data contained 11,952 complete cases (1,002 in the TANIA study, 10,687 in the MARIANNE study, and 263 in the Batman study), that is, valid EQ-5D-3L values and valid FACT-B scores, including subscores. Six observations from male patients in the MARIANNE study were excluded. Table 1 presents the summary statistics for this sample. The average age was 52.61 ± 11.5 years. Patients spanned the full spectrum of disease measured by the EQ-5D-3L and FACT-B. Figure 1 shows the distribution of the EQ-5D-3L reported in the sample. The distribution is multimodal, has a spike of observations at full health, and displays the gap between full health and the next feasible state. There are 3295 observations (27.6% of the sample) at full health and 172 (1.4% of the sample) with values worse than death (<0). Figure 1 also shows the FACT-B distribution, which spans the entire feasible range (0–123) and is positively skewed.

**Table 1:** Descriptive statistics

**Variable (n = 11,952)**	**Mean ± SD**	**Minimum**	**Maximum**
EQ-5D-3L index score	0.762 ± 0.227	−0.594	1
Mobility	1.243 ± 0.438	1	3
Self-care	1.120 ± 0.340	1	3
Usual activities	1.370 ± 0.529	1	3
Pain/discomfort	1.611 ± 0.545	1	3
Anxiety/depression	1.492 ± 0.556	1	3
FACT-B	40.204 ± 20.269	0	123
Physical well-being	5.434 ± 4.829	0	33
Social/family well-being	6.394 ± 5.290	0	28
Emotional well-being	6.407 ± 4.522	0	24
Functional well-being	9.663 ± 6.073	0	28
BCS	12.306 ± 6.234	0	38
Age (y)/10	5.261 ± 1.150	2.2	8.7

BCS, breast cancer–specific; EQ-5D-3L, three-level EuroQol five-dimensional questionnaire; FACT-B, Functional Assessment of Cancer Therapy—Breast Cancer.

### SUR Ordered Probit Models

**Fig. 2:**
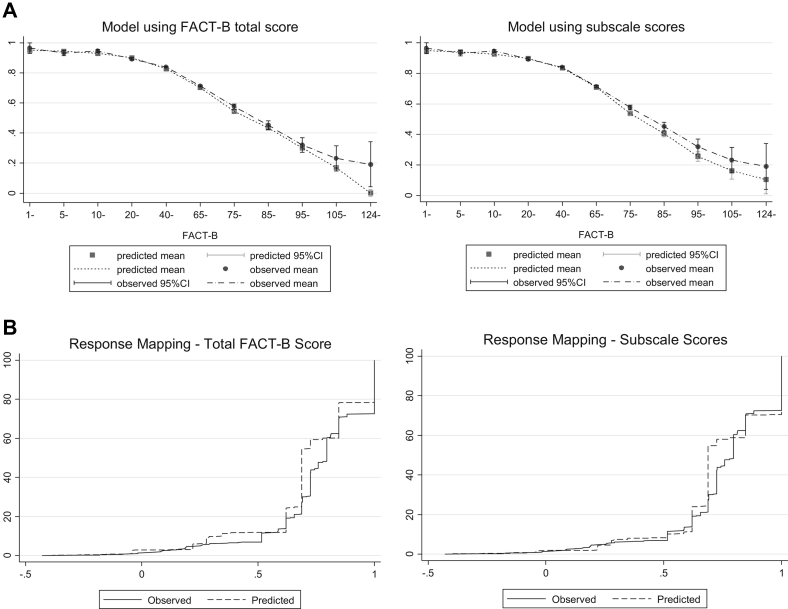
(A) Mean predicted vs. mean observed values using response mapping with total FACT-B score and subscale scores. (B) Cumulative distribution functions using response mapping with total FACT-B score and subscale scores. CI, confidence interval; FACT-B, Functional Assessment of Cancer Therapy—Breast Cancer.

We estimated two response mappings to the EQ-5D-3L using SUR ordered probit models: the first using the total FACT-B score and the second using the FACT-B subscale scores. Table 2 presents the model fit statistics. Of these models, the mean error, MAE, and RMSE are smaller in the model that includes the subscales of FACT-B. AIC and BIC are also lower in the subscale model, and the proportions predicted at full health and in a state worse than death are also closer to the observed data when using the subscales. Figure 2 displays mean predicted versus mean observed values for response mapping using the total FACT-B score and the FACT-B subscale scores. Using the subscale model improves fit at higher values of FACT-B when patients are in poorer health. Figure 2 also shows the CDFs for the SUR response mappings using the total FACT-B score and the FACT-B subscale scores. The figure shows that the model that includes the individual subscale scores of FACT-B has a better fit than the model that includes only the FACT-B summary score.

**Table 2:** Model fit for response mappings and ALDVMMs

**FACT-B total/subscales**	**Number of components**	**Log likelihood**	**AIC**	**BIC**	**Mean error**	**Mean absolute error**	**RMSE**	**Proportion of predicted observations at full health (%)**	**Proportion of predicted observations below 0 (%)**
*Response mapping*									
Total scores	–	−29,869.95	59,809.91	60,068.53	0.0117	0.1341	0.1824	21.7	2.8
Subscales	–	−27,530.01	55,170.02	55,576.43	0.0092	0.1216	0.1682	29.6	1.89

*ALDVMMs*									
Total FACT-B	1	−735.2737	1,480.547	1,517.493	0.0018	0.1330	0.1829	34.3	0.2
	2	1,217.156	−2,406.312	−2,302.863	0.0006	0.1324	0.1815	25.2	1.6
	3	1,395.884	−2,745.768	−2,575.818	−0.000007	0.1318	0.1815	27.4	1.5
	4	1,724.382	−3,384.763	−3,148.310	0.0001	0.1332	0.1818	27.4	1.5
Subscales	1	435.7411	−853.4822	−786.9798	−0.0002	0.1207	0.1656	33.9	0.1
	2	2,476.873	−4,901.747	−4,709.629	0.0005	0.1199	0.1654	25.9	1.6
	3	2,867.129	−5,648.258	−5,330.524	−0.0004	0.1155	0.1631	27.6	1.4
	4	3,075.795	−6,031.590	−5,588.240	−0.00009	0.1158	0.1633	27.0	1.5

*Note*. Proportion of observations at full health = 27.6%, proportion in a state worse than death = 1.4%. Note that AIC and BIC are not comparable between response mapping and ALDVMMs.

AIC, Akaike information criterion; ALDVMM, adjusted limited dependent variable mixture model; BIC, Bayesian information criterion; FACT-B, Functional Assessment of Cancer Therapy—Breast Cancer; RMSE, root mean square error.

### Adjusted Limited Dependent Variable Mixture Model

We estimated ALDVMMs with one to four components and using FACT-B subscale scores versus total FACT-B score both within the components and as determinants of component membership. We attempted to estimate models using five components, but there were problems with convergence. Table 2 presents the fit statistics.

**Fig. 3:**
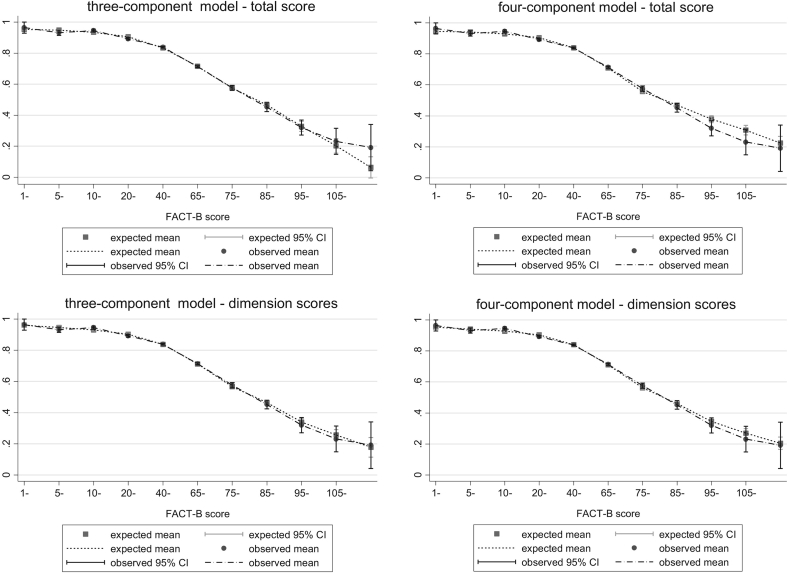
Mean predicted vs. mean observed values using three- and four-component ALDVMMs with total FACT-B score and subscale scores. ALDVMM, adjusted limited dependent variable mixture model; CI, confidence interval; FACT-B, Functional Assessment of Cancer Therapy—Breast Cancer.

**Fig. 4:**
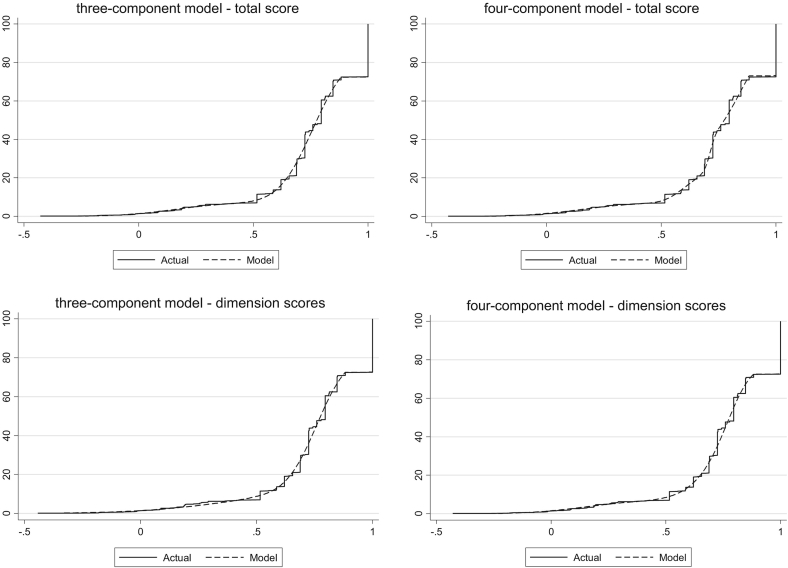
Cumulative distribution functions using three- and four-component ALDVMMs with FACT-B subscale scores. ALDVMM, adjusted limited dependent variable mixture model; FACT-B, Functional Assessment of Cancer Therapy—Breast Cancer.

Of the models estimated that include the total FACT-B score, the model with four components is preferred when looking at the AIC and BIC. Nevertheless, the three-component model has lower error measures. Both the three- and four-component models are more accurate than models with fewer components in simulating the proportion of patients at full health and in a state worse than death. Figure 3 shows mean predicted versus mean observed values in the three- and four-component ALDVMMs. The three-component model appears to predict the observed data better than the four-component model at most of the FACT-B scores. Nevertheless, the three-component model predicts less well individuals scoring extremely poor values on the FACT-B scale. Figure 4, which displays the CDFs for the mixture models that use three and four components, shows that simulated data from the mixture models fit the observed data closely for both models that use the total FACT-B score. Given that, the evidence suggests that both the three- and four-component models that use the total FACT-B score fit the data very closely. Because there is little visible difference in model fit for the two models (Fig. 3, Fig. 4) as well as the model fit statistics, we prefer the simpler three-component model with fewer parameters.

From Table 2 it can be seen that of the models estimated that include the FACT-B subscale scores in place of the total FACT-B score, the model with three components is preferred when looking at the AIC and BIC as well as the mean error. Nevertheless, the four-component model has lower MAE and RMSE. The three-component subscale model produces the most accurate simulation of the data in terms of the proportion predicted at full health and in a state worse than death. Figure 3 shows little difference between the model fits of the three- and four-component models when using the subscale scores. Figure 4 shows that simulated data from both the three- and four-component mixture models using subscale scores fit the observed data closely. We again consider the three-component model as the preferred model to avoid adding unnecessary parameters.

The results show that including individual FACT-B subscales as independent variables improves model fit compared with using the total FACT-B score. MAE and RMSE as well as AIC and BIC are consistently smaller in the models that include individual subscales. Figure 3 displays the mean predicted versus mean observed values for the mixture models. It shows that models including the individual subscale scores of FACT-B are preferred to those that use the total FACT-B score. Figure 4 shows that all ALDVMMs produce simulated data that fit the observed data very closely.

Model fit statistics in Table 2 suggest that, in all cases, ALDVMMs outperform response mapping using SUR ordered probit models. Similarly, Fig. 3, Fig. 4 show that the ALDVMMs provide a significant improvement in model fit to the response mappings when compared with Figure 2. In particular, Figure 4 shows that the ALDVMMs produce simulated data that better represent the observed data particularly at the low and high extremes of the EQ-5D-3L. The mixture models are better equipped to deal with the EQ-5D-3L distribution than is the response mapping. More specifically, the ALDVMMs are generally better able to fit the EQ-5D-3L distribution in patients with poor health states.

There is little difference between the model fit statistics of the ALDVMM when using total FACT-B score compared with the response mapping using individual FACT-B subscale scores (see Table 2). This is supported by the figures that show no obvious superiority of the response mapping using FACT-B subscale scores compared with using the ALDVMM with total FACT-B score. The ALDVMM improves model fit, particularly in the main part of the distribution compared with response mapping, regardless of whether the total FACT-B score or individual subscale scores are used.

In each of the subscale models estimated, the BCS subscale was found to have a strong and statistically significant influence. The response mapping showed that this subscale has a strong influence in predicting the pain/discomfort and self-care dimensions of the EQ-5D-3L. The ALDVMM results show that besides having some significant influence on the within-component estimates of the EQ-5D-3L, the BCS subscale of the FACT-B has a substantial influence on the component membership in these models.

The ALDVMMs estimated in this study can be easily implemented via Excel files and Stata’s .ster files, which are provided in the Supplemental Materials found at https://doi.org/10.1016/j.jval.2018.06.006.

## Discussion

Little is known about the relationship between FACT-B and PBMs for use in the economic evaluation of technologies in breast cancer. The only such study identified in the HERC Database of Mapping Studies [2] is based on data from patients in Singapore and it used simplistic mapping methods [15]. The study found that linear regression outperformed other simple mapping models based on narrow assessment of summary fit. The lack of previous mapping studies in this disease area emphasizes the need for more research in utility mapping in patients with breast cancer.

This article provides reliable estimates for the calculation of the EQ-5D-3L as a function of FACT-B in patients with breast cancer. We provide estimates that can be used when analysts have access only to total FACT-B scores or when they have access to subscale responses. The mapping algorithms produced in this study should not be used to map from FACT-B to any other PBM, including the EQ-5D-5L.

Our results show that although response mapping has been previously shown to be superior to direct utility mapping using, for example, linear regression and generalized linear models [5], the bespoke ALDVMM offers a better fit than response mapping using SUR ordered probit models in this setting. This adds to the growing literature on the performance of mixture models for mapping and is consistent with those that find they outperform response mapping [4].

We find that models that use the subscales of FACT-B as independent variables perform the best. Nevertheless, subscale scores are not always reported in summary reports of clinical studies. Often, the total FACT-B score will be all that is available and therefore the corresponding summary score models are important for analysts populating cost-effectiveness models. We find that when using the ALDVMM to map the total FACT-B score to the EQ-5D-3L, model fit is similar to the response mapping, which includes each of the subscales.

For the reasons discussed here, we suggest that the three-component ALDVMM that uses the individual subscales is the preferred model with the best overall fit to the data and should be used when individual subscale scores are available. When individual subscale scores are not available, the three-component ALDVMM using the total FACT-B score should be used.

Consistent with other mapping studies, we found age to be important and statistically significant in predicting the EQ-5D-3L. This is particularly important when mapping algorithms are used in cost-effectiveness analysis. Sex, which is usually included in mapping studies, is not included here because the samples were all female.

Our findings show that the BCS subscale of FACT-B significantly predicts the EQ-5D-3L. This suggests that it is important to include the BCS questions, either in the form of the FACT-B subscale or within the total FACT-B score. Other FACT measures or instruments that do not contain any BCS questions should not be used to map to PBMs in patients with breast cancer. For example, a general cancer instrument, FACT-G, has previously been used to map to the EQ-5D-3L in a range of patients with cancer [15], [16], [17], [18]. Our results show that more specific cancer instruments will produce more accurate results.

This study has limitations. The data used contain repeated observations on the same patients over time. To account for this we used clustered standard errors; nevertheless, this was not found to significantly change the results. Although the data used in this study span the full range of the FACT-B and the EQ-5D-3L scores, there are a few observations at the higher end of the FACT-B score. This could be an artefact of the clinical study populations, but may be typical of the breast cancer population. It is important that analysts consider the relevant population for the specific health technology in question when using these results in cost-effectiveness analysis.

## Conclusions

The ALDVMM has been shown to outperform linear regression, response mapping, and other statistical models in a range of disease settings. Further research into different types of mixture models, for example, a beta-based mixture model [19], could help to further develop mapping methods in patients with breast cancer.

## Supplemental Materials

EQ-5D FACT-B calculator2

Out of Sample Predictions_FACT-B

mappingFACTB

## Footnotes

Supplementary data associated with this article can be found in the online version at https://doi.org/10.1016/j.jval.2018.06.006.
